# Shock Wave Treatment in Composite Tissue Allotransplantation

**Published:** 2011-09-15

**Authors:** Christian Andreas Radu, Jurij Kiefer, Dominik Horn, Martin Rebel, Eva Koellensperger, Martha Maria Gebhard, Henning Ryssel, Guenter Germann, Matthias Artur Reichenberger

**Affiliations:** ^a^Department of Hand, Plastic and Reconstructive Surgery, Burn Center, BG Trauma Center Ludwigshafen, Plastic and Hand Surgery of the University of Heidelberg, Heidelberg, Germany; ^b^Department of Pathology, Klinikum Ludwigshafen, Germany; ^c^Clinic for Plastic and Reconstructive Surgery, ETHIANUM, Heidelberg, Germany; ^d^Department of Experimental Surgery, University of Heidelberg, Germany

## Abstract

**Introduction:** Composite tissue allotransplantation is a newly emerged field of transplantation. Shock wave technology has already been used in the treatment of urologic and orthopedic disorders. Recent studies demonstrated a suppression of the early proinflammatory immune response. **Methods:** 50 allogeneic hindlimb transplantations were performed on rats in 5 different groups. Group A (n = 10), (Lewis → Brown-Norway) received 500 impulses of extracorporeal shock wave. Groups B, C, D, and E served as control groups with group B (n = 10) receiving no immunosuppression, group C (n = 10) receiving FK506 and prednisolone, group D (n = 10) receiving no immunosuppression with isograft transplantations (Brown-Norway → Brown-Norway) and group E receiving 500 impulses of extracorporeal shock wave on the contralateral hindlimb. **Results:** Rejection of the allogeneic hindlimb occurred on average 7.12 days after transplantation in group A (extracorporeal shock wave). Rejection was significantly delayed compared to the control groups B (no immunosuppression) and E (contralateral hindlimb), where rejection of the allogeneic hindlimb occurred on average 5.49 and 5.6 days after transplantation (*t* test, *P* < .01). No rejection was seen in groups C and D. **Conclusions:** For the first time, shock waves have been applied in a composite tissue allotransplantation model and resulted in a significant immunosuppressive effect. These promising first results have showed that shock wave treatment is clinically relevant in composite tissue allotransplantation and justify subsequent research to improve the experimental and clinical outcome.

Composite tissue allotransplantation (CTA) is an emerging field of transplantation that offers a potential treatment for complex tissue defects after traumatic loss or tumor resection and for the repair of congenital abnormalities. Since CTA of hand, face, and other tissues is a clinical reality, CTA has gained importance as an alternative reconstruction procedure. However, all these procedures are not routinely performed because most CTAs compared to solid organ transplantation are non—life saving operations. Consequently, there has been a wide debate about whether the benefit of CTA justifies the risks of lifelong immunosuppressive therapies, as they remain nonspecific to the type of donor and still bear significant risks of serious side effects. These side effects include an increased incidence of neoplasms, organ toxicity, and opportunistic infections.

The most appealing solution to this problem would be the induction of immunologic tolerance, defined as lifelong, donor-specific unresponsiveness without the need for immunosuppressive drugs.

In the past 10 years, it has been shown that it is possible to achieve prolonged hand allograft survival with a good functional outcome, superior to that obtained by prostheses, with mainly the same triple drug immunosuppressive regime used routinely in kidney transplants to control rejection of transplanted hands.^1^^-^8 These intermediate long-term results have far exceeded expectations both from an immunological and a functional point of view. Improvement of immunotherapy with the eventual aim of tolerance induction therefore continues to be a highly desirable goal in both organ and CTA.

Until now, the effect of extracorporeal shock waves (ESW) has not been investigated in a CTA. There is very limited published information as to the safety and complications of ESW treatment in conjunction with CTA.

Shock waves are high-energy acoustic waves, generated through an electrohydraulic method in this case, that result from high voltage explosion and vaporization.^9^^,^^10^ Since its successful introduction in 1980 for fragmentation of kidneys stone, ESW has been adapted for many other clinical indications such as musculoskeletal disorders (nonunion of long bone fractures, calcifying tendonitis).^11^^-^14 Previous animal and clinical studies found that during shock wave treatment various angiogenic factors are being released.^15^^-^26 Aicher et al^27^ demonstrated that low-energy ESW treatment improves recruitment of circulating endothelial progenitor cells via enhanced expression of chemoattractant factors in hindlimb ischemia in a rat model. Recently published studies showed that early proangiogenic and anti-inflammatory effects of ESW promote tissue revascularization and wound healing by augmenting angiogenesis and suppressing proinflammatory immune response.^28^^,^^29^ The potential of ESW to augment angiogenesis and suppress proinflammatory immune response has aroused interest about its use in other procedures. The aim of this study was to investigate the effect of ESW in a rodent in vivo model of CTA (hindlimb transplantation).

## MATERIAL AND METHODS

### Animals

Male Lewis (LW) (RT 1l) rats (Charles River WIGA, Sulzfeld, Germany) (160-180 g, 7-11 weeks old) were used as hindlimb donors. Brown-Norway (BN) (RT 1n) rats (Charles River WIGA, Sulzfeld, Germany) (220-240 g, 11-15 weeks old) served as recipients. This combination represents a very strong histocompatibility mismatch.

The experimental protocol was approved by a review committee of the state of Baden-Wuerttemberg, Germany, in accordance to the guidelines of the German Animal Welfare Act. Surgical procedures were performed under standard aseptic conditions.

### In vivo transplantation model

In all experiments anaesthesia was induced and maintained by an intraperitoneal injection of sodium pentobarbital (50 mg/kg body weight) (Narcoren; Merial GmbH, Germany) and an intramuscular injection of ketamine hydrochloride (30 mg/kg body weight) (Ketanest S; Parke-Davis GmbH, Berlin, Germany). After initial shaving, the left hindlimb of the donor rat was prepared under aseptic conditions. Skin, muscle, and bone were cut at mid thigh level, the femoral vessels were first dissected in continuity with ligation of branching vessels. Heparin (5000 U) was injected into the penile vein. The femoral artery was cannulated and the hindlimb perfused with ringer solution containing 2 U/mL heparin for 10 minutes. Thereafter the femoral vessels were divided.

The hindlimbs of the BN rats were amputated in the way described earlier. The donor and recipient femurs were then joined by the use of a 1.0 or 1.2 mm intramedullary K-wire. The femoral artery and vein were anastomosed using standard microsurgical techniques with 10-0 nylon sutures. After anastomosis, the ventral and dorsal muscle groups of the thigh were approximated and the skin was sutured. Ischemia time was approximated at 2 hours. Postoperatively, the recipients received 5 mL of 0.9% NaCl subcutaneously for fluid substitution. As a pain medication, buprenorphine (Temgesic; Schering-Plough, Kenilworth, New Jersey) 0.03 mg/kg was applied intramuscularly (IM) twice a day for 10 days postoperatively.

During the operation, the core temperature of the recipient BN rat and the hindlimb temperature were closely monitored rectally as well as by an intramuscular temperature probe. The muscle temperature was kept between 28°C and 33°C throughout the procedure. The rats' core temperatures were kept between 34°C and 37.5°C using a heating pad and lamp.

### Shock waves

**Figure 1:**
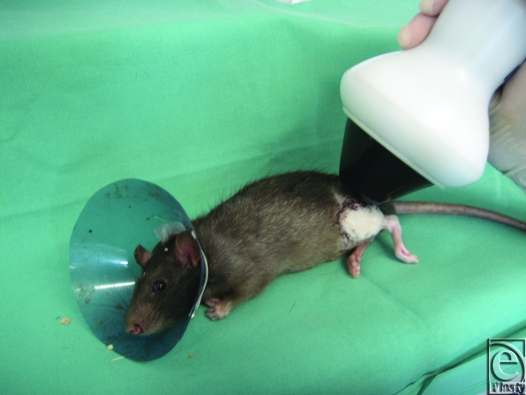
Immediately after wound closure animals in the ESW treatment group (group A) were exposed to 500 impulses at 0.19 mJ/mm^2^ (dermaPACE; SANUWAVE Inc, Alpharetta, Georgia) to the entire hindlimb.

Immediately after wound closure, animals in the ESW treatment groups (groups A and E) were exposed to 500 impulses ESW treatment at 0.19 mJ/mm^2^ (dermaPACE; SANUWAVE Inc, Alpharetta, Georgia) to the entire transplanted hindlimb (group A) and the contralateral hindlimb (group E). As a contact medium, we used ultrasound transmission gel between the ESW apparatus and the rat's skin (Fig 1).

### Experimental model

Allogeneic hindlimb transplantations from BN to LEW were carried out in 50 animals. Animals were randomly divided into 1 of the 5 experimental groups:

Group A (n = 10), (LW → BN) received ESW locally with 500 impulses at 0.19 mJ/mm^2^ immediately after wound closure. Groups B, C, D, and E served as control groups with group B (n = 10) receiving no immunosuppression, group C (n = 10) receiving FK506 (Prograft, Astellas Pharma, Munich, Germany) 0.1 mg/kg daily IM and Prednisolone (Solu-Decortrin H; Merck Pharma, Darmstadt, Germany) 0.2 mg/kg daily IM for 100 days, group D (n = 10) receiving no immunosuppression with isograft transplantations (BN → BN), and group E (n = 10) (LW) → BN) receiving ESW immediately after transplantation on the contralateral hindlimb with 500 impulses at 0.19 mJ/mm^2^.

### Postoperative monitoring

The rats were clinically assessed for their general and hindlimb condition every 8 hours by 2 surgeons. Body weight was measured 3 times daily. To avoid autocannibalism protective collars made of plastic film were applied to all animals. The rats were followed up until 100 days postoperatively or until rejection of the hindlimb occurred. Rats in a poor general condition or with self-inflicted bites were terminated.

In the hindlimb, we assessed color, edema, skin condition, hair condition, and consistency of the thigh of the hindlimb. Edema was classified into 5 grades (grade 0 = no edema, grade 4 = maximal edema). Consistency on palpation ranged from grade 0 (soft = normal) to grade 4 (leather-like). Clinical hindlimb rejection was defined as the sequence of a sudden edema (grades 1-2) within 8 hours on the dorsum of the foot, a subsequent change in color away from pink and hardening of the thigh (grades 2-3). All 3 parameters needed to be present for the definition of rejection. Both observers had to define rejection independently. Edema of the thigh, epidermal loss, desquamation, hair loss, or blistering were not used for the definition of the onset of rejection.

### Histology

Besides assessing the timing of rejection by clinical observation, standardized skin and muscle biopsies (Musculus tibialis anterior) were taken separately from the lower leg on day 5, 10, 20, 50, and 100 or when clinical rejection was suspected. We developed a histological score called the N2R-Score that defines 4 grades of necrosis, infiltration of inflammatory cells and regeneration.

The necrosis was described in a semiquantitative manner with 0 showing no signs of necrosis, 1 showing less than 50% of necrosis, 2 showing between 50% and 90% of necrosis and 3 showing 90% and 100% of necrosis. Infiltration of inflammatory cells such as granulocytes, macrophages, and mesenchymal proliferation was also graduated in 4 different grades with 0 showing no infiltration, 1 showing few infiltration, 2 showing moderate infiltration, and 3 showing dense infiltration of inflammatory cells. Graduation of regeneration of either skin or muscle was also assessed in 4 different grades with grade 0 showing no signs of regeneration, 1 showing less than 10% of regeneration with vital tissue, 2 showing between 10% and 50% of regeneration, and 3 showing more than 50% of regeneration.

**Figure 2:**
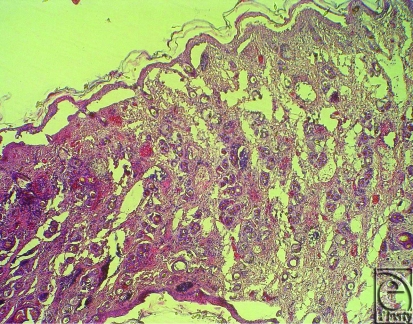

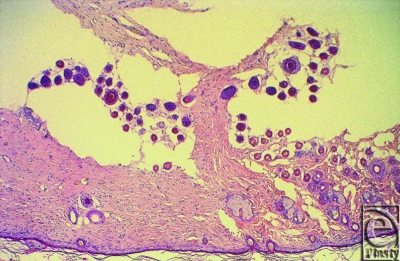
(*a*) Representative images of histological skin analysis (group B) on day 5 after allogeneic hindlimb transplantation with approximately 20% necrosis (grade 1), moderate infiltration of inflammatory cells (grade 2), and 0% regeneration (grade 0) (N2R-score). (*b*) Representative images of histological skin analysis (group D) on day 50 after hindlimb transplantation with no necrosis (grade 0), few infiltration of inflammatory cells (grade 1), and less than 10% of regeneration (grade 1) (N2R-score).

Histological rejection was defined as when either necrosis or infiltration of inflammatory cells rose to grade 2 and the other parameter rose to grade 1 (Fig 2a). Even though regeneration as the third parameter was always seen in the control groups, it did not help reliably for assessing the onset of histological rejection (Fig 2b). In accordance with the literature, histological rejection was first seen in skin biopsies because skin is known to be the most immunogenic component in CTA.^30^

### Animal protection and statistics

All in vivo experiments were performed according to the German Law on the Protection of Animals.

Data are given as the mean ± standard deviation (SD). Data were tested for normal distribution using Shapiro-Wilk test and Kolmogorov-Smirnov test. Clinical data were tested for significance using unpaired *t* test, whereas histopathological data were tested using paired *t* test. Level of significance was determined at α = 0.05. All tests were performed by using SAS software (SAS Institute, Inc, Cary, North Carolina).

## RESULTS

In group A (n = 10) (ESW), hindlimb rejection occurred on average on day 7.12 (SD = 0.83). In detail, rejection occurred once on day 5, three times on day 7, four times on day 7.3, and twice on day 8.

In group B (n = 10) (no immunosuppression) hindlimb rejection occurred on average on day 5.49 (SD = 0.70). In detail, rejection occurred once on day 4, twice on day 5, twice on day 5.3, four times on day 6, and once on day 6.3. In group C (n = 10) (FK506/prednisolone) and group D (n = 10) (isograft) no hindlimb rejection occurred until termination of the follow-up on day 100. In group E (ESW to contralateral hindlimb), hindlimb rejection occurred on average on day 5.6 (SD = 0.53). In detail, rejection occurred twice on day 5, three times on day 5.3, twice on day 5.7, twice on day 6, and once on day 6.7.

**Figure 3:**
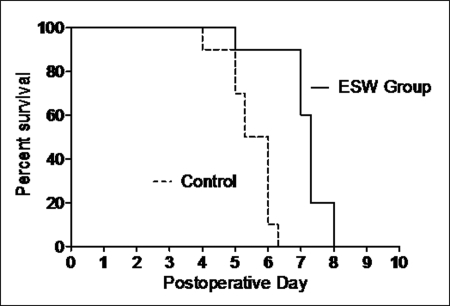
Timing of the clinical rejection after allogeneic hindlimb transplantations. Group A (n = 10) received ESW locally, group B (n = 10) served as a control group receiving no immunosuppression. The mean rejection/survival time is shown in days postoperatively. The difference is significant between group A and group B (*t* test < 0.01).

Comparing group A and B rejection was statistically significantly delayed using ESW treatment (unpaired *t* test, *P* < .01) (Fig 3, Table 1). There was no significant difference comparing rejection between the control groups B and E (unpaired *t* test, *P* > .9).

Histological rejection based on the N2R-Score was seen in all skin and muscle biopsies taken when clinical rejection was suspected. The results of the biopsies taken on day 5 showed rejection only in one animal in group A, 5 animals in group B, and 5 animals in group E. No histological rejection was seen in group C and D.

The histological findings therefore supported the clinical findings and showed that rejection was significantly delayed using ESW treatment (paired *t* test, *P* < .01).

## DISCUSSION

Improving immunosuppressive therapy with the eventual goal of tolerance is a major goal not only in CTA but in all organ transplantations. Over 10 years, after the first allogeneic hand transplantation overall graft and patient survival as well as functional outcome is very promising.^2^^,^^4^^,^^5^^,^^8^^,^^31^^-^34 One graft failure in hand transplantations was reported to be due to noncompliance.^4^^,^^35^ One fatality has been reported following combined hand and face transplantation that occurred from overwhelming sepsis presumably due to the immunosuppressive protocol.^36^^,^^37^

Still the lifelong use of immunosuppressive drugs is associated with numerous complications including delayed wound healing, opportunistic infections, drug-related toxicities, skin malignancies, low-grade lymphomas, lymphoproliferative disorders, and end-organ toxicity.^38^

For the first time, we applied shock waves in a microsurgical in vivo model of CTA. The aim of this study was to further investigate immunosuppressive therapies in a vascularized model of CTA by administering ESW. We were able to show that ESW treatment significantly prolongs allograft survival compared to the control groups. Shockwave treatment to the contralateral hindlimb did not prolong allograft survival and underlined the direct effect of ESW on the allograft. Several studies have demonstrated the efficiency of ESW in the treatment of bone fractures and tendon healing.^14^^,^^15^^,^^24^^,^^25^ Furthermore ESW treatment has been shown to contribute to complete wound healing in a variety of wounds, including burns, ischemic, traumatic, and postoperative wounds.^20^^,^^22^^,^^39^^,^^40^ While the exact mechanism of ESW remains unclear, several studies state that ESW serves as mechanotransduction and improves blood supply by vasodilatation at early stage and neovascularization at a late stage, associated with an increased expression of angioactive factors such as Nitrogen monoxide (NO) and Vascular Endothelial Growth Factor (VEGF). Recent results of animal and clinical studies suggest that ESW treatment suppresses early proinflammatory immune response. In a skin flap model in rodents, Kuo et al^41^ stated that there has been a positive effect in reducing the ischemic zones of flaps by increasing tissue perfusion. In their study, histological analysis of the flap tissue demonstrated that the inflammatory cell infiltration was attenuated immediately after ESW treatment compared with that in control group.^41^ In a subsequent study, Davis et al observed a global suppression of proinflammatory factors in a severe, full-thickness and highly inflammatory cutaneous burn wound in a murine model 4 and 24 hours after a single ESW treatment. This observation was coupled with impaired neutrophil and macrophage migration and a subsequent suppression of proinflammatory cytokines (IL-1β, IL-6, and TNFα).^28^

We hypothesize that ESW is capable of modulating specific T-cell response (either directly or via a B-cell mediated pathway) with a decrease of acute early proinflammatory mediators (chemokines, cytokines, and metalloproteinases). Moreover, we assume that ESW improves blood supply postoperatively with expression of angioactive factors in the operative tissue that provides the minimum requirement of oxygen and nutrition for ischemic tissue to survive.

Although our results have shown a significant immunosuppressive effect in a rather complex model of CTA, the immunosuppressive effect is still limited. After our evaluation this may be due to different facts: One certainty is that in CTA compared to organ transplantations different tissue types such as skin, muscles, tendons, and bones are being transplanted, with skin being known to be the most immunogenic component.^5^^,^^42^ Furthermore, there may be limitations regarding the interpretation of the data due to the experimental design. First, for better understanding of the mechanisms of ESW treatment involved in CTA, additional molecular studies should be examined at earlier time points to clarify the improvement in tissue survival. Second, our decision for selecting one single ESW treatment was an empirical decision, primarily based on our previous flap surgery studies and recommendations by the manufacturer.^43^^-^45

This is the first study with application of ESW treatment in CTA. These first promising results of ESW administration in CTA raise several questions and require further research to clarify dose-effect relations, long-term effects of ESW on immune and reparative cells and the changes in cell populations. We believe that ESW can then be used as an adjunct to either a tolerance model or immunosuppressive model of CTA transplantation rather than as a single agent. While clinically ESW is often preformed under anaesthesia, PACE technology ESW treatment does not require anaesthesia, so it may easily allow more applications postoperatively as a continuum of care if it is determined that a protocol of ESW is beneficial to CTA.

Further investigations with repeat ESW treatments to see whether multiple interventions would be beneficial in terms of prolonged graft survival and ESW treatment being added to different low-dose immunosuppressive protocols in small animal studies are ongoing and in development.

## Figures and Tables

**Table 1:** Statistical figures

Group	A	B	C	D	E
n	10	10	10	10	10
Rejection	7.12 ± 0.83	5.49 ± 0.70	100	100	5.6 ± 0.53
Days of rejection in detail	5/7/7/7/	4/5/5/5.3/	No rejection	No rejection	5/5/5.3/5.3/
	7.3/7.3/7.3/	5.3/6/6/6/			5.3/5.7/5.7/
	7.3/8/8	6/6.3			6/6/6.7

## References

[1] Gazarian A, Abrahamyan DO, Petruzzo P (2007). [Hand allografts: experience from Lyon team]. Ann Chir Plast Esthet.

[2] Lanzetta M, Petruzzo P, Dubernard JM (2007). Second report (1998-2006) of the International Registry of Hand and Composite Tissue Transplantation. Transpl Immunol.

[3] Madani H, Hettiaratchy S, Clarke A, Butler PE (2008). Immunosuppression in an emerging field of plastic reconstructive surgery: composite tissue allotransplantation. J Plast Reconstr Aesthet Surg.

[4] Petruzzo P, Lanzetta M, Dubernard JM (2010). The International Registry on Hand and Composite Tissue Transplantation. Transplantation.

[5] Ravindra KV, Buell JF, Kaufman CL (2008). Hand transplantation in the United States: experience with 3 patients. Surgery.

[6] Ravindra KV, Wu S, McKinney M, Xu H, Ildstad ST (2009). Composite tissue allotransplantation: current challenges. Transplant Proc.

[7] Whitaker IS, Duggan EM, Alloway RR (2008). Composite tissue allotransplantation: a review of relevant immunological issues for plastic surgeons. J Plast Reconstr Aesthet Surg.

[8] Wu S, Xu H, Ravindra K, Ildstad ST (2009). Composite tissue allotransplantation: past, present and future—the history and expanding applications of CTA as a new frontier in transplantation. Transplant Proc.

[9] Gerdesmeyer L, Maier M, Haake M, Schmitz C (2002). [Physical-technical principles of extracorporeal shockwave therapy (ESWT)]. Orthopade.

[10] Ogden JA, Toth-Kischkat A, Schultheiss R (2001). Principles of shock wave therapy. Clin Orthop Relat Res.

[11] Chaussy C, Schuller J, Schmiedt E, Brandl H, Jocham D, Liedl B (1984). Extracorporeal shock-wave lithotripsy (ESWL) for treatment of urolithiasis. Urology.

[12] Eisenberger F, Chaussy C (1978). Contact-free renal stone fragmentation with shock waves. Urol Res.

[13] Wang CJ (2003). An overview of shock wave therapy in musculoskeletal disorders. Chang Gung Med J.

[14] Wang CJ, Huang HY, Chen HH, Pai CH, Yang KD (2001). Effect of shock wave therapy on acute fractures of the tibia: a study in a dog model. Clin Orthop Relat Res.

[15] Chen YJ, Wang CJ, Yang KD (2004). Extracorporeal shock waves promote healing of collagenase-induced Achilles tendinitis and increase TGF-beta1 and IGF-I expression. J Orthop Res.

[16] De Sanctis MT, Belcaro G, Nicolaides AN (2000). Effects of shock waves on the microcirculation in critical limb ischemia (CLI) (8-week study). Angiology.

[17] Fukumoto Y, Ito A, Uwatoku T (2006). Extracorporeal cardiac shock wave therapy ameliorates myocardial ischemia in patients with severe coronary artery disease. Coron Artery Dis.

[18] Huemer GM, Meirer R, Gurunluoglu R (2005). Comparison of the effectiveness of gene therapy with transforming growth factor-beta or extracorporal shock wave therapy to reduce ischemic necrosis in an epigastric skin flap model in rats. Wound Repair Regen.

[19] Kuo YR, Wang CT, Wang FS, Chiang YC, Wang CJ (2009). Extracorporeal shock-wave therapy enhanced wound healing via increasing topical blood perfusion and tissue regeneration in a rat model of STZ-induced diabetes. Wound Repair Regen.

[20] Meirer R, Brunner A, Deibl M, Oehlbauer M, Piza-Katzer H, Kamelger FS (2007). Shock wave therapy reduces necrotic flap zones and induces VEGF expression in animal epigastric skin flap model. J Reconstr Microsurg.

[21] Meirer R, Huemer GM, Oehlbauer M, Wanner S, Piza-Katzer H, Kamelger FS (2007). Comparison of the effectiveness of gene therapy with vascular endothelial growth factor or shock wave therapy to reduce ischaemic necrosis in an epigastric skin flap model in rats. J Plast Reconstr Aesthet Surg.

[22] Oi K, Fukumoto Y, Ito K (2008). Extracorporeal shock wave therapy ameliorates hindlimb ischemia in rabbits. Tohoku J Exp Med.

[23] Stojadinovic A, Elster EA, Anam K (2008). Angiogenic response to extracorporeal shock wave treatment in murine skin isografts. Angiogenesis.

[24] Wang CJ, Huang HY, Pai CH (2002). Shock wave-enhanced neovascularization at the tendon-bone junction: an experiment in dogs. J Foot Ankle Surg.

[25] Wang CJ, Wang FS, Yang KD (2003). Shock wave therapy induces neovascularization at the tendon-bone junction. A study in rabbits. J Orthop Res.

[26] Yan X, Zeng B, Chai Y, Luo C, Li X (2008). Improvement of blood flow, expression of nitric oxide, and vascular endothelial growth factor by low-energy shockwave therapy in random-pattern skin flap model. Ann Plast Surg.

[27] Aicher A, Heeschen C, Sasaki K, Urbich C, Zeiher AM, Dimmeler S (2006). Low-energy shock wave for enhancing recruitment of endothelial progenitor cells: a new modality to increase efficacy of cell therapy in chronic hind limb ischemia. Circulation.

[28] Davis TA, Stojadinovic A, Anam K (2009). Extracorporeal shock wave therapy suppresses the early proinflammatory immune response to a severe cutaneous burn injury. Int Wound J.

[29] Kuo YR, Wang CT, Wang FS, Yang KD, Chiang YC, Wang CJ (2009). Extracorporeal shock wave treatment modulates skin fibroblast recruitment and leukocyte infiltration for enhancing extended skin-flap survival. Wound Repair Regen.

[30] Horner BM, Ferguson KK, Randolph MA (2010). In vivo observations of cell trafficking in allotransplanted vascularized skin flaps and conventional skin grafts. J Plast Reconstr Aesthet Surg.

[31] Knobloch K, Rennekampff HO, Meyer-Marcotty M, Gohritz A, Vogt PM (2009). [Organ transplantation, composite tissue allotransplantation, and plastic surgery]. Chirurg.

[32] Ravindra KV, Wu S, Bozulic L, Xu H, Breidenbach WC, Ildstad ST (2008). Composite tissue transplantation: a rapidly advancing field. Transplant Proc.

[33] Siemionow M, Bozkurt M, Kulahci Y (2007). Current status of composite tissue allotransplantation. Handchir Mikrochir Plast Chir.

[34] Swearingen B, Ravindra K, Xu H, Wu S, Breidenbach WC, Ildstad ST (2008). Science of composite tissue allotransplantation. Transplantation.

[35] Kanitakis J, Jullien D, Petruzzo P (2003). Clinicopathologic features of graft rejection of the first human hand allograft. Transplantation.

[36] Hui-Chou HG, Nam AJ, Rodriguez ED (2010). Clinical facial composite tissue allotransplantation: a review of the first four global experiences and future implications. Plast Reconstr Surg.

[37] Meningaud JP, Benjoar MD, Hivelin M, Hermeziu O, Toure G, Lantieri L (2010). The procurement of total human face graft for allotransplantation: a preclinical study and the first clinical case [published online ahead of print June 15, 2010]. Plast Reconstr Surg.

[38] Baumeister S, Kleist C, Dohler B, Bickert B, Germann G, Opelz G (2004). Risks of allogeneic hand transplantation. Microsurgery.

[39] Haupt G, Chvapil M (1990). Effect of shock waves on the healing of partial-thickness wounds in piglets. J Surg Res.

[40] Meirer R, Kamelger FS, Piza-Katzer H (2005). Shock wave therapy: an innovative treatment method for partial thickness burns. Burns.

[41] Kuo YR, Wu WS, Hsieh YL (2007). Extracorporeal shock wave enhanced extended skin flap tissue survival via increase of topical blood perfusion and associated with suppression of tissue pro-inflammation. J Surg Res.

[42] Radu CA, Bosch N, Bauer TM (2007). Immunosuppressive effect of tryptophan metabolites in composite tissue allotransplantation. Plastic and reconstructive surgery.

[43] Reichenberger MA, Keil H, Mueller W Optimal timing of extracorporeal shock wave treatment to protect ischemic tissue [published online ahead of print May 16, 2011]. Ann Plast Surg.

[44] Reichenberger MA, Keil H, Mueller W (2011). Comparison of extracorporal shock wave pretreatment to classic surgical delay in a random pattern skin flap model. Plast Reconstr Surg.

[45] Reichenberger MA, Germann G, Roth HJ, Meirer R, Engel H (2009). Preoperative shock wave therapy reduces ischemic necrosis in an epigastric skin flap model. Ann Plast Surg.

